# Radiomics Analysis of Computed Tomography helps predict poor prognostic outcome in COVID-19

**DOI:** 10.7150/thno.46428

**Published:** 2020-06-05

**Authors:** Qingxia Wu, Shuo Wang, Liang Li, Qingxia Wu, Wei Qian, Yahua Hu, Li Li, Xuezhi Zhou, He Ma, Hongjun Li, Meiyun Wang, Xiaoming Qiu, Yunfei Zha, Jie Tian

**Affiliations:** 1 College of Medicine and Biomedical Information Engineering, Northeastern University, Shenyang, Liaoning, 110819, China.; 2 Beijing Advanced Innovation Center for Big Data-Based Precision Medicine, School of Medicine and Engineering, Beihang University, Beijing, 100191, China.; 3 Department of Radiology, Renmin Hospital of Wuhan University, Wuhan 430060, China.; 4 Department of medical imaging, Henan Provincial People's Hospital; People's Hospital of Zhengzhou University; People's Hospital of Henan University; Zhengzhou, Henan, 450003, China.; 5 Department of Electrical and Computer Engineering, University of Texas at El Paso, 500 West University Avenue, El Paso, TX, 79968, United States.; 6 Department of Radiology, Huangshi Central Hospital, Affiliated Hospital of Hubei Polytechnic University, Edong Healthcare Group, Hubei, 435000, China; 7 Department of Radiology, Beijing Youan Hospital, Capital Medical University, Beijing, 100069, China.; 8 Engineering Research Center of Molecular and Neuro Imaging of Ministry of Education, School of Life Science and Technology, Xidian University, Xi'an, Shaanxi, 710126, China.; 9 CAS Key Laboratory of Molecular Imaging, Institute of Automation, Chinese Academy of Sciences, Beijing, 100190, China.

**Keywords:** COVID-19, Computed tomography, Radiomics, Prognosis, Poor outcome

## Abstract

**Rationale:** Given the rapid spread of COVID-19, an updated risk-stratify prognostic tool could help clinicians identify the high-risk patients with worse prognoses. We aimed to develop a non-invasive and easy-to-use prognostic signature by chest CT to individually predict poor outcome (death, need for mechanical ventilation, or intensive care unit admission) in patients with COVID-19.

**Methods:** From November 29, 2019 to February 19, 2020, a total of 492 patients with COVID-19 from four centers were retrospectively collected. Since different durations from symptom onsets to the first CT scanning might affect the prognostic model, we designated the 492 patients into two groups: 1) the early-phase group: CT scans were performed within one week after symptom onset (0-6 days, n = 317); and 2) the late-phase group: CT scans were performed one week later after symptom onset (≥7 days, n = 175). In each group, we divided patients into the primary cohort (n = 212 in the early-phase group, n = 139 in the late-phase group) and the external independent validation cohort (n = 105 in the early-phase group, n = 36 in the late-phase group) according to the centers. We built two separate radiomics models in the two patient groups. Firstly, we proposed an automatic segmentation method to extract lung volume for radiomics feature extraction. Secondly, we applied several image preprocessing procedures to increase the reproducibility of the radiomics features: 1) applied a low-pass Gaussian filter before voxel resampling to prevent aliasing; 2) conducted ComBat to harmonize radiomics features per scanner; 3) tested the stability of the features in the radiomics signature by several image transformations, such as rotating, translating, and growing/shrinking. Thirdly, we used least absolute shrinkage and selection operator (LASSO) to build the radiomics signature (RadScore). Afterward, we conducted a Fine-Gray competing risk regression to build the clinical model and the clinic-radiomics signature (CrrScore). Finally, performances of the three prognostic signatures (clinical model, RadScore, and CrrScore) were estimated from the two aspects: 1) cumulative poor outcome probability prediction; 2) 28-day poor outcome prediction. We also did stratified analyses to explore the potential association between the CrrScore and the poor outcomes regarding different age, type, and comorbidity subgroups.

**Results:** In the early-phase group, the CrrScore showed the best performance in estimating poor outcome (C-index = 0.850), and predicting the probability of 28-day poor outcome (AUC = 0.862). In the late-phase group, the RadScore alone achieved similar performance to the CrrScore in predicting poor outcome (C-index = 0.885), and 28-day poor outcome probability (AUC = 0.976). Moreover, the RadScore in both groups successfully stratified patients with COVID-19 into low- or high-RadScore groups with significantly different survival time in the training and validation cohorts (all *P* < 0.05). The CrrScore in both groups can also significantly stratify patients with different prognoses regarding different age, type, and comorbidities subgroups in the combined cohorts (all *P* < 0.05).

**Conclusions:** This research proposed a non-invasive and quantitative prognostic tool for predicting poor outcome in patients with COVID-19 based on CT imaging. Taking the insufficient medical recourse into account, our study might suggest that the chest CT radiomics signature of COVID-19 is more effective and ideal to predict poor outcome in the late-phase COVID-19 patients. For the early-phase patients, integrating radiomics signature with clinical risk factors can achieve a more accurate prediction of individual poor prognostic outcome, which enables appropriate management and surveillance of COVID-19.

## Introduction

An ongoing outbreak of coronavirus disease 2019 (COVID-19) associated with a novel coronavirus SARS-CoV-2 is spreading across the world 1,2. As of May 8, 2020, more than 3 million people have been confirmed, and more than 200 thousand have died around the world 3. The high infection rate of COVID-19 poses a great strain on medical resources, especially critical care resources in hospitals 4-6. Given the rapid spread of COVID-19, an updated risk-stratify prognostic tool might help clinicians identify the high-risk patients with worse prognoses.

Many attempts have been made to identify the risk factors to predict prognosis in COVID-19. The prognostic risk factors included clinical factors (e.g., sex, age, smoking status, and underlying diseases 4,7), laboratory examination (e.g., lymphocyte count, C-reactive protein (CRP), lactic dehydrogenase 8,9), and computed tomography (CT) score derived from chest CT features 10-12. However, most published studies converted prognostic analysis into binary classification, such as predicting the probability of discharging hospital after ten days 13, predicting the probability of progression to severe/critical/mortal states 14-17. Very few models were built for continuous prognostic prediction 18,19, which can enable us to acquire the prognostic hazard for each patient, and predict the prognostic situation for each patient at any time point.

Radiomics, as a quantitative analytic tool, can quantify imaging phenotypes by extracting specific features from medical image modalities 20,21. This novel method exhibited potential in assessing treatment response and predicting survival outcomes 22-24. Recent studies showed that chest CT imaging has an important role not only in the diagnosis of COVID-19 but also in prognostic prediction 25,26. Some research suggested identifying COVID-19 patients into different stages based on the radiological findings on CT imaging, such as ground-glass opacity (GGO) and consolidation 27-29. During the course of illness, the frequency of GGO and consolidation, the predominant pattern, and the involvement of lung lobes changed over time on the chest CT, indicating the prognosis of COVID-19 patients 30,31. However, whether a quantitative prognostic tool by using CT radiomics features could assess and predict prognosis in patients with COVID-19 remains unclear.

Therefore, we aimed to develop a non-invasive and easy-to-use prognostic signature by CT images to individually predict patients with poor outcome (death, need for mechanical ventilation, or intensive care unit (ICU) admission) that need critical care and close monitoring.

## Materials and Methods

### Patients and Follow-up

The institutional review board of Renmin Hospital of Wuhan University (Centre 1), Huangshi Central Hospital (Centre 2), Henan Provincial People's Hospital (Centre 3), and Beijing Youan Hospital (Centre 4) approved this multi-regional retrospective study, and the informed consent was waived.

From November 29, 2019 to February 19, 2020, a total of 492 patients diagnosed with COVID-19 by etiological evidence of reverse transcriptase-polymerase chain reaction (RT-PCR) test were retrospectively collected. To relieve the impact of different durations from symptom onsets to the first CT scanning, we designated the 492 patients into two groups: 1) the early-phase group: CT scans were performed within one week after symptom onset (0-6 days, n = 317); and 2) the late-phase group: CT scans were performed one week later after symptom onset (≥7 days, n = 175). Here, day 0 was defined as the initial day of symptom onset, which was self-reported by patients on admission.

In the early phase group: 212 patients from Center 1 (n = 106) and Center 2 (n = 106) comprised the training cohort since they all came from Hubei Province (the hardest-hit region). 105 patients from Center 3 (n = 65) and Center 4 (n = 40) comprised the validation cohort. In the late-phase group: 139 patients from Center 1 (n = 125) and Center 2 (n = 14) comprised the training cohort, and 36 patients from Center 3 (n = 23) and Center 4 (n = 13) comprised the validation cohort. All the included patients had regular follow-up for at least five days. The end-points of this study was the poor outcome, which was defined as death, need for mechanical ventilation, or ICU admission 6,32,33. The follow-up durations were assessed from CT evaluation to poor outcome.

Baseline information for each patient, including age, sex, type on admission, comorbidity, follow-up duration, and poor outcome status at the last follow-up was collected. Baseline information is shown in **Table 1**. The type of COVID-19 was defined based on the diagnosis and treatment protocols from the National Health Commission of the People's Republic of China (trial version 7) 34. In this study, we simplified the mild and common types into the mild type, and severe and critical type into the severe type for analysis convenience 6.

### Image acquisition and lung volume segmentation

**Figure 1:**
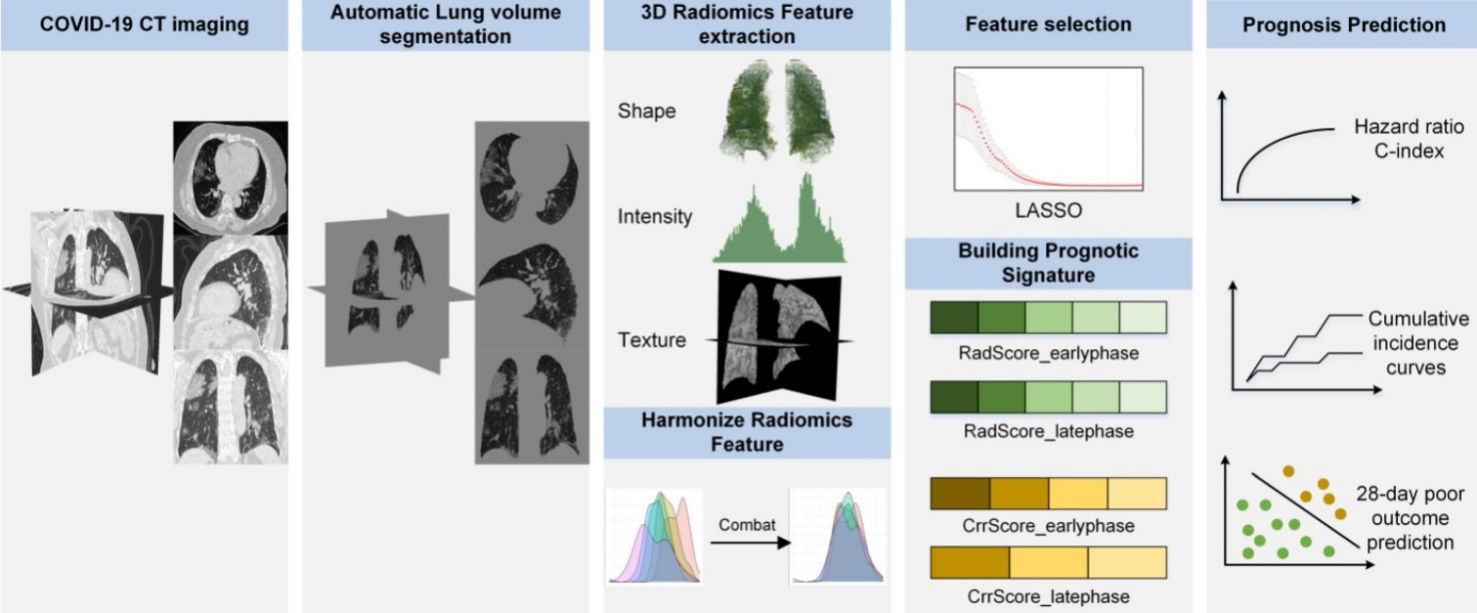
** Radiomics framework of predicting the poor prognostic outcome in patients with COVID-19.** RadScore_earlyphase and RadScore_latephase means the radiomics signature built for predicting poor outcome in the early-phase and late-phase COVID-19 patients, respectively. CrrScore_earlyphase and CrrScore_latephase means the clinic-radiomics signature built for predicting poor outcome in the early-phase and late-phase COVID-19 patients, respectively.

In this study, all patients underwent non-contrast enhanced chest CT on admission. CT scanning parameters are in Supplementary Methods 1. Since many COVID-19 infections manifested as bilateral or even total lung involvement on chest CT imaging 35, we extracted the whole lung volume for radiomics analysis (**Figure 1**) and defined it as 3D-ROI. We built a DenseNet121-FPN model to segment lung volume automatically, and this model is pre-trained using 1.4 million natural images 36, and fine-tuned on VESSEL12 dataset 37 (details in Supplementary Methods 2).

### Features extraction and radiomics signature building

After segmenting lung volume automatically, we limited the intensities to *I*_mean_ ± 3*I*_std_ (*I*_mean_, mean of image intensity; and *I*_std_, standard deviation of image intensity) to minimize the influence of voxel distribution and contrast variation on the following feature extraction. Afterward, we conducted a low-pass Gaussian filter to prevent aliasing and increase the reproducibility of radiomics features 38,39, and then resampled CT images into 3 mm × 3 mm × 3 mm voxel spacing to reduce the impact of different equipment and scanning parameters. Finally, we used a bin-width of 25 Hounsfield units to discretize and followed the IBSI guideline 40 to extract 3D radiomic features by PyRadiomics version 2.20 41, and standardized radiomic features by z-score normalization. In order to reduce the multicenter effect for the radiomics features, we conducted ComBat 42 to harmonize radiomics features per scanner.

### Radiomics signature building

A total of 107 3D radiomic features were extracted to describe the pulmonary information from following aspects: 1) shape feature (n=14): they described the pulmonary morphological change in patients with COVID-19; 2) first-order features (n=18): they reflected the imaging intensity change in lung volume; 3) texture feature (n=75): they quantified the degree of voxel change and the relationship between pneumonia area and non-pneumonia area in microscopic-level.

Despite the various pulmonary information quantified by the radiomic features, not all of them were related to the poor outcome in COVID-19. Consequently, we used the least absolute shrinkage and selection operator (LASSO) 43 based on Cox proportional hazards regression to select potential features that are associated with poor outcome. We used 5-fold cross-validation in the training cohort to choose the optimal parameters and avoid over-fitting. We built a radiomic signature (RadScore) by the corresponding coefficients. The RadScore built in the early-phase group is defined as RadScore_earlyphase, and the RadScore built in the late-phase group is defined as RadScore_latephase.

To test the stability of the features in the signature, we conducted the following three transformations 39 on the original image and mask: 1) rotation: both images and masks were rotated by a random degree, which ranged from 0° to 2°; 2) translation: both images and masks were translated with a random fraction, which ranged from 0 to 0.2; 3) volume adaptation: the 3D-ROI mask were grown and shrunk to alter the volume by a random fraction, which ranged from 0 to 0.1. Rotation and translation are used to emulate the different patient positioning, and volume adaptation is used to mimic variance in the boundaries of the 3D-ROI. The stability was assessed by intraclass correlation coefficient (ICC). The 95% confidence interval (CI) of the ICC larger than 0.90 means the feature is robust.

### Clinical model and clinic-radiomics signature building

Since the recovered patient leads to the censoring of the poor outcome in the Kaplan-Meier method, we used Fine-Gray competing risk regression to assess the cumulative incidence of poor outcome 44,45, aiming to explore the prognostic value of clinical risk factors and radiomics signature in COVID-19. As recovery is not completely independent from the poor outcome, recovery without evidence of death, mechanical ventilation, or ICU admission was treated as a competing event. (The recovery criteria are in the Supplementary Methods 3). Specifically, we included four candidate clinical risk factors (age, sex, type on admission, and comorbidity), and used backward stepwise with minimum AIC to select risk factors to build a clinical model.

Since the RadScore can describe pulmonary function from CT imaging and clinical features can reflect patients' condition from clinical aspects, we further incorporated the RadScore and the clinical risk factors into the competing risk regression analysis, and used minimum AIC criteria to select the final model. The clinic-radiomics signature was built by measuring the competing risk regression score (CrrScore) for each patient. The CrrScore was defined as the linear combination of risk factors with corresponding coefficients. The CrrScore built in the early-phase group is defined as CrrScore_earlyphase, and the CrrScore built in the late-phase group is defined as CrrScore_latephase. A higher CrrScore means high-risk of death, need for mechanical ventilation or ICU admission, and relatively shorter survival time of poor outcome, and vice visa.

### Statistical analysis

All statistical analyses were performed with R software (version 3.5.1). The statistical difference of clinical variables was assessed with the unpaired, 2-tailed chi-square test or t-test for categorical or continuous variables, respectively.* P* < .05 indicated a statistically significant difference. The potential association of the RadScore and CrrScore with poor outcome was first assessed in the training cohort and then validated in the independent validation cohort by using cumulative incidence curves and hazard ratio (HR). Patients were allocated into low- or high-RadScore/CrrScore groups through the median of RadScore/CrrScore. We estimated cumulative incidence curves for each group and used Gray's test to assess the difference between the two curves. The performance of the clinical model, RadScore, and CrrScore was estimated by the concordance index (C-index), which can measure the concordance between the predicted poor outcome and the actual poor outcome (0.5 means poor concordance and 0.7 indicates good concordance). Receiver-operating characteristic (ROC) analysis was applied to assess the performance of the three prognostic signatures (clinical model, RadScore, and CrrScore) in predicting 28-day poor outcome. Stratified analyses were performed to explore the potential association between the CrrScore and the poor outcomes regarding different age, type, and comorbidity subgroups in the combined cohorts.

## Results

### Patient characteristics

A total of 492 patients with COVID-19 were included from four regions, including Wuhan China. As of the last follow-up, 40 patients (8.1%) had experienced a confirmed poor outcome in the total cohort. The median (interquartile range [IQR]) follow-up of poor outcome in the total cohort is 13 (9-18) days. In the early phase group, the median follow-up of poor outcome was 13 (9-17) days for the training cohort, 15 (11-20) days for the validation cohort. In the late phase group, the median follow-up of poor outcome was 12 (8-15) days for the training cohort, 11 (9-18) days for the validation cohort.

### Radiomics signature construction and validation

A total of 5 features were selected for constructing RadScore_earlyphase (**Figure S1**), and the formula was as follows: -0.3300× shape_Sphericity - 0.1605 × glcm_ClusterShade + 0.1529 × glcm_Correlation + 0.3426 × glrlm_LongRunHighGrayLevelEmphasis - 0.1068 × ngtdm_Complexity.

A total of 5 features were selected for constructing RadScore_latephase, and the formula was as follows: -0.3749×shape_Flatness - 0.4955 × shape_Sphericity - 0.0484 × firstorder_10Percentile - 0.0653 × firstorder_Minimum + 0.3023 × ngtdm_Complexity.

We also measured the time to derive the radiomics features for each individual, which took 17.6±0.4 s on a machine with an Intel Core i7-7700 CPU and 16 GB memory.

After transforming the image and masks by rotation, translation, and volume adaption, all the features in the RadScore exhibited 95% CI of ICC larger than 0.9, indicating the features in the RadScore are robust.

**Figure 2:**
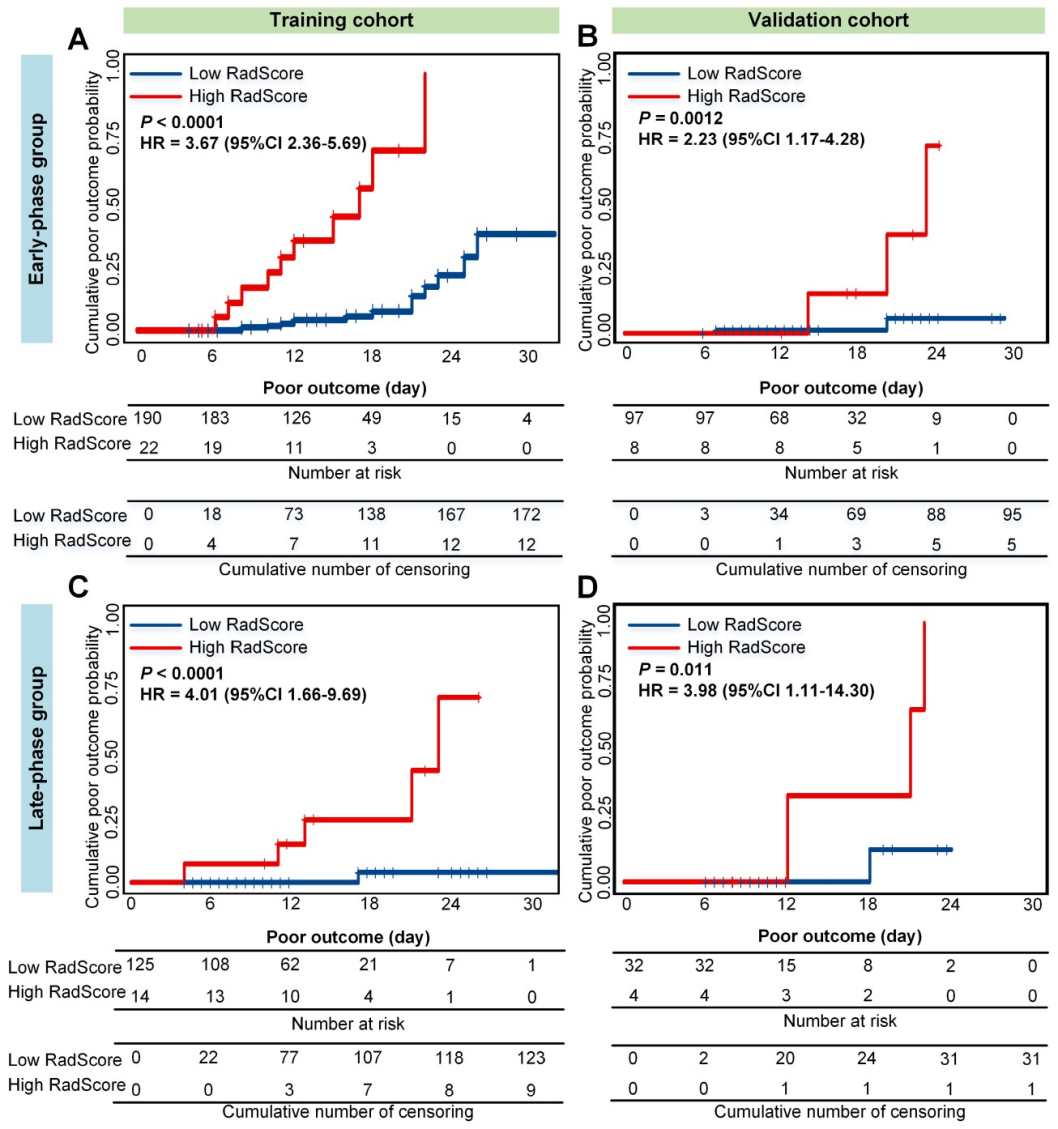
** Cumulative poor outcome probability according to the risk strata defined by RadScore.** A) and C) in the training cohort; B) and D) in the validation cohort. A) and B) assess the cumulative probability in the early-phase group. C) and D) assess the cumulative probability in the late-phase group.

Accordingly, patients were allocated into a low- or high-RadScore group by the median of RadScore in the training cohort. As shown in **Figure 2**, in the early-phase group, higher RadScores were significantly associated with higher cumulative probability of poor outcome (HR = 3.67 (2.36-5.69), *P* < 0.0001 in the training cohort; and HR = 2.23 (1.17-4.28), *P* = 0.0012 in the validation cohort). Similar results were observed in predicting cumulative probability of poor outcome in the late-phase group (HR = 4.01 (1.66-9.69),* P* < 0.0001 in the training cohort; and HR = 3.98 (1.11-14.30), *P* = 0.011 in the validation cohort).

Moreover, the RadScore also showed good performance on poor outcome prediction in the training cohort (C-index = 0.758 (0.619-0.897) in the early-phase group; C-index = 0.886 (0.702-0.999) in the late-phase group). The prognostic performance of the RadScore was further confirmed in the validation cohort (C-index = 0.752 (0.600-0.906) in the early-phase group; C-index = 0.885 (0.718-0.999) in the late-phase group).

### Performance of the clinical model

The univariate analyses of clinical risk factors are shown in Figure S2 and S3. In the early-phase group, three factors (age, type, and comorbidity) exhibited significant prognostic value (all *P* < 0.05), and were selected to build the clinical model. It yielded a C-index of 0.708 (0.6569-0.848) and 0.793 (0.679-0.907) in the training and validation cohort, respectively. In the late-phase group, none factors showed significant prognostic value (all *P* > 0.05). The clinical model was built by incorporating type and age, and yielded a C-index of 0.697 (0.509-0.886) and 0.793 (0.534-0.944) in the training and validation cohort, respectively.

### Clinic-radiomics signature construction and validation

We used backward stepwise competing risk regression with the minimum AIC to select risk factors and built a clinic-radiomics signature (CrrScore); the formulas are shown in **Table 2**. In the early-phase group, the CrrScore that incorporated the RadScore and the three clinical risk factors (age, comorbidity, and type) showed significant improvement in the training cohort when compared with the clinical model (C-index = 0.826 (0.714-0.937), *P* = 0.012 in the training cohort; and C-index = 0.850 (0.763-0.935), *P* = 0.45 in the validation cohort; **Table 3**). In the late-phase group, the CrrScore that incorporated the RadScore and the two clinical risk factors (age and type) showed significant improvement when compared with the clinical model (C-index = 0.911 (0.796-0.999), *P* = 0.029 in the training cohort; and C-index = 0.886 (0.675-0.999), *P* < 0.01 in the validation cohort).

**Figure 4:**
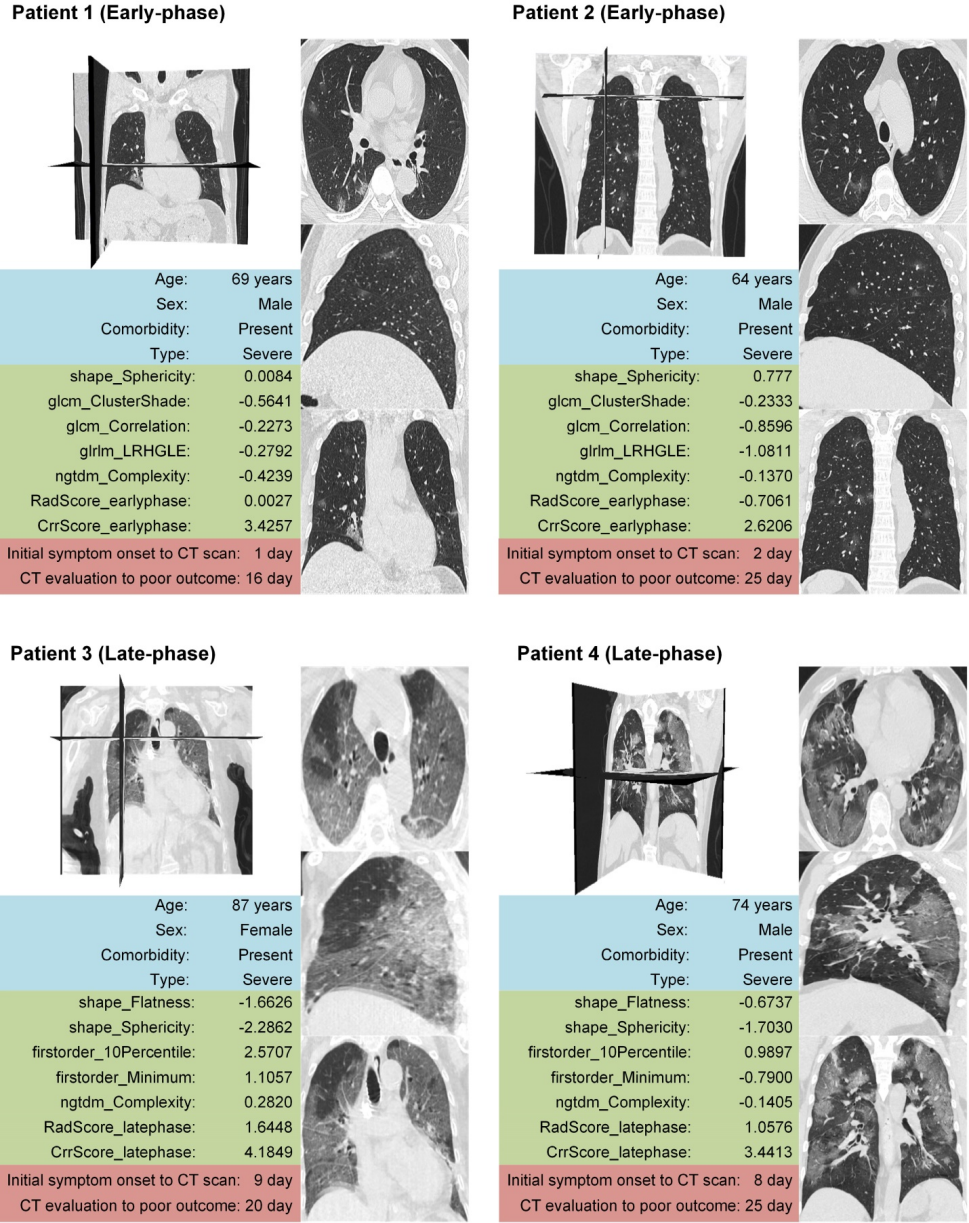
** Four representative clinical high-risk patients with older age, severe type, comorbidities and different prognoses.** The patient with relatively higher RadScore and CrrScore had shorter time to reach the poor outcome. Patient 1 and 2 were in the early-phase group (the time interval between initial symptom onset and the CT scan < 7 days). Patient 3 and 4 were in the late-phase group (the time interval between initial symptom onset and the CT scan ≥ 7 days).

**Figure 5:**
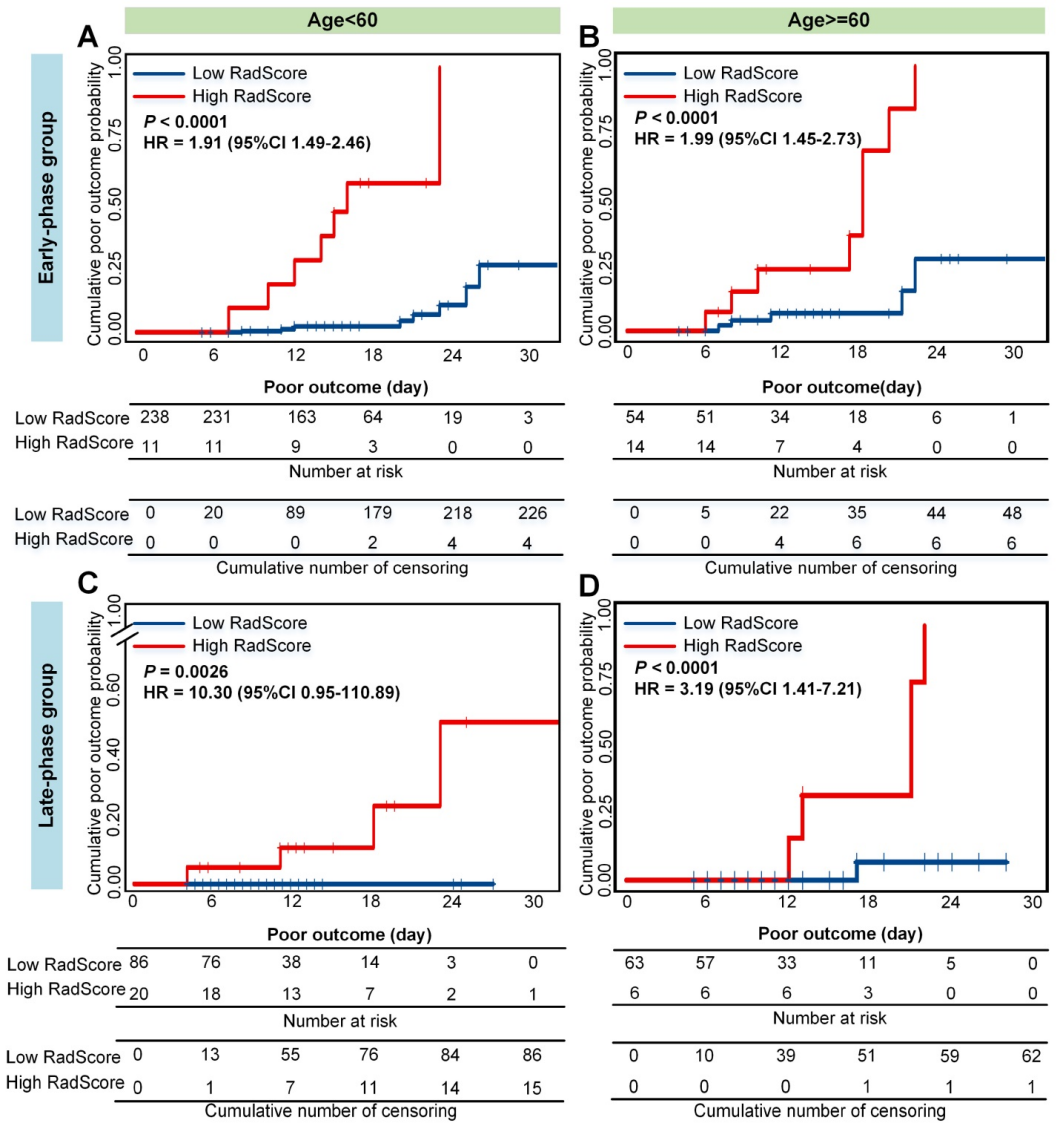
** Cumulative poor outcome probability according to the risk strata defined by CrrScore within age subgroup in the combined cohorts.** A) and C) for the age < 60 subgroup; B) and D) for the age ≥ 60 subgroup. A) and B) assess the cumulative probability in the early-phase group. C) and D) assess the cumulative probability in the late phase group.

**Figure 6:**
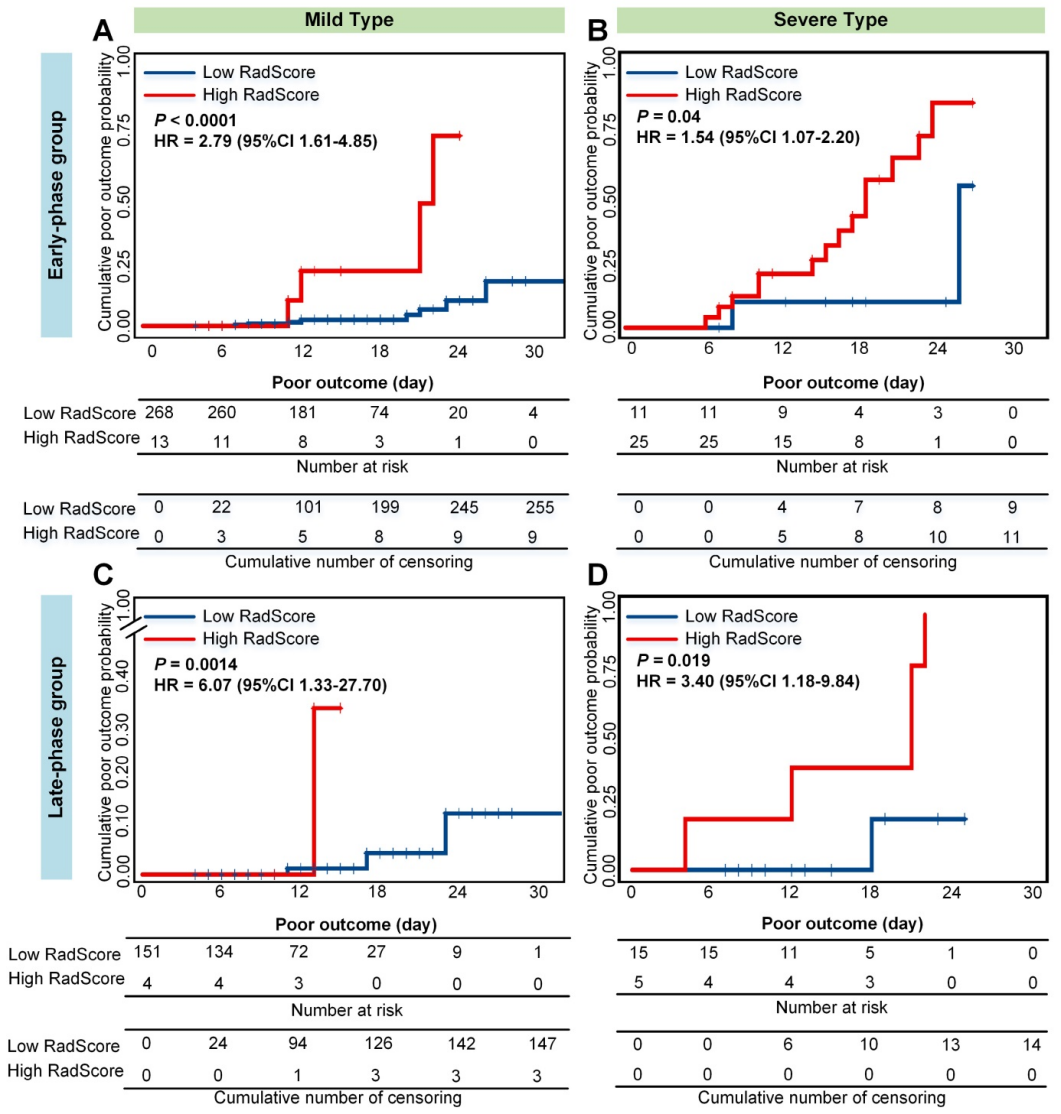
** Cumulative poor outcome probability according to the risk strata defined by CrrScore within type subgroup in the combined cohorts.** A) and C) for the mild type subgroup; B) and D) for the severe type subgroup. A) and B) assess the cumulative probability in the early-phase group. C) and D) assess the cumulative probability in the late-phase group.

**Figure 7:**
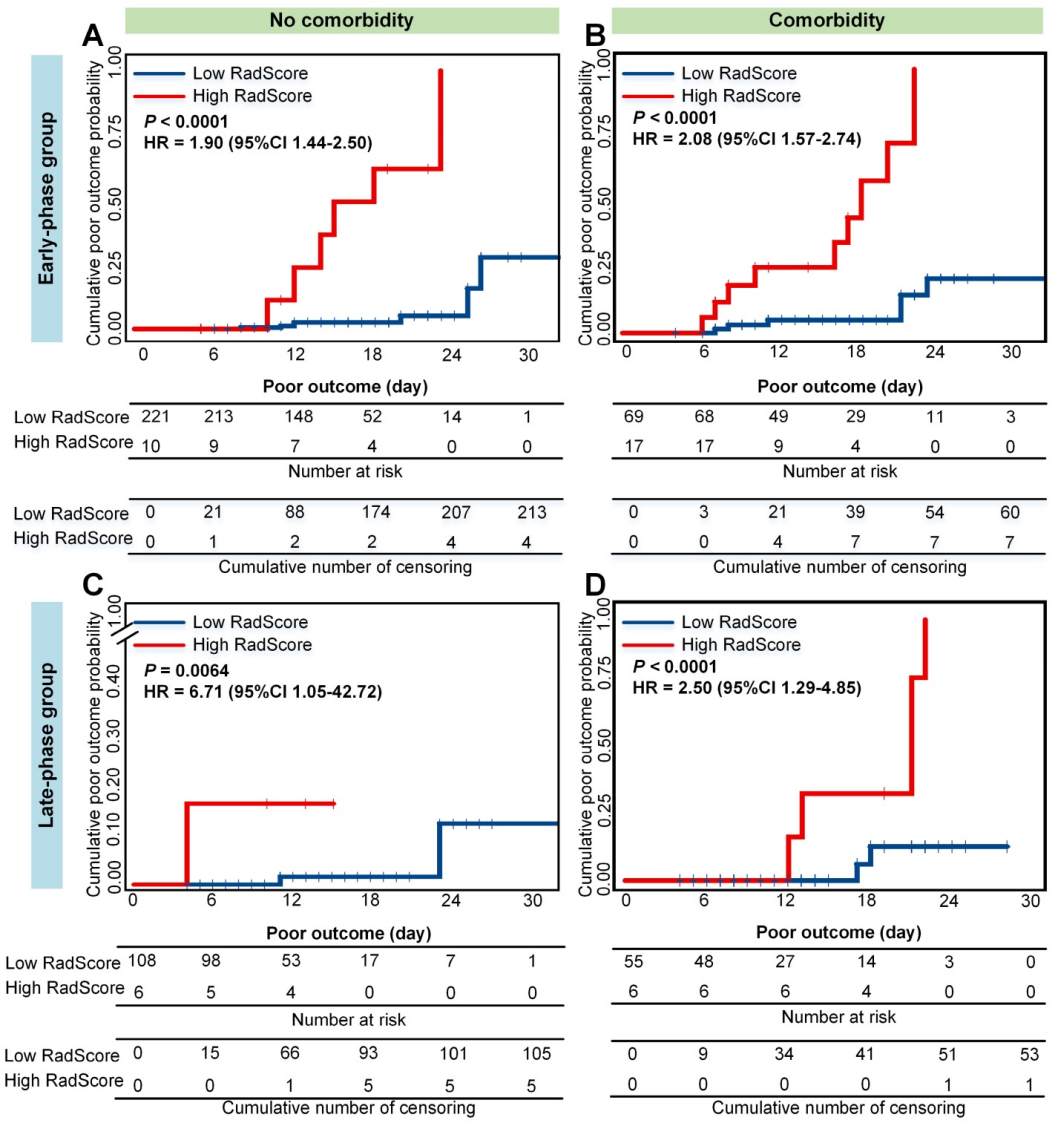
** Cumulative poor outcome probability according to the risk strata defined by CrrScore within comorbidity subgroup in the combined cohorts.** A) and C) for the non-comorbidity subgroup; B) and D) for the comorbidity subgroup. A) and B) assess the cumulative probability in the early-phase group. C) and D) assess the cumulative probability in the late-phase group.

Older and severe patients with comorbidities were reported at a high risk of death 4,46. Therefore, we performed stratified analysis regarding different age, type, and comorbidity in the combined cohorts. As shown in **Figure 5, 6 and 7**, CrrScore can stratify patients with different prognoses of poor outcome (all *P* < 0.05) within age, type, and comorbidity subgroups in the combined cohorts. Moreover, the distribution of CrrScore showed significant differences between the different age, type, and comorbidity subgroups (Figure S4, all *P* < 0.001). **Figure 4** exhibited four representative clinical high-risk patients with older age, severe type, and comorbidities. The patient with relatively higher RadScore and CrrScore had a shorter time to reach the poor outcome.

### 28-day poor outcome prediction

In predicting the 28-day poor outcome probability among the early-phase COVID-19 patients, the clinical model achieved an AUC of 0.748 (0.638-0.858) and 0.685 (0.410-0.960), the RadScore yielded an AUC of 0.752 (0.631-0.873) and 0.816 (0.614-0.999) in the training cohort and validation cohort, respectively. The CrrScore showed significant improved performance in the training cohort compared with the clinical model (AUC = 0.855 (0.740-0.920), *P* = 0.015 in the training cohort; and AUC = 0.862 (0.680-0.999),* P* = 0.28 in the validation cohort). In predicting the 28-day poor outcome probability among the late-phase COVID-19 patients, and the RadScore and CrrScore demonstrated similar performance in the validation cohort with AUC of 0.885 (0.718-0.999) and 0.886 (0.675-0.999), respectively.

## Discussion

In this study, we assessed CT-derived radiomics features and clinical risk factors in predicting the poor outcomes (death, need for mechanical ventilation, or ICU admission) in patients with COVID-19. Due to the various durations from symptom onsets to the first CT scanning, we designated patients into the early-phase group (CT scans were performed 0-6 days after the symptom onset), and the late-phase group (CT scans were performed ≥7 days after symptom onset).

In the early-phase group, the clinical model and radiomics signature (RadScore) demonstrated comparable performance in poor outcome estimation. The clinic-radiomics signature (CrrScore), which combined the radiomics signature (RadScore) and clinical risk factors, showed improvement in estimating poor outcome (C-index increased from 0.752 to 0.850), and predicting probability of 28-day poor outcome (AUC increased from 0.816 to 0.862). This indicated that RadScore might have complementary value to the clinical prognostic factors in finding out the high-risk patients with poor outcome in the early-phase COVID-19 patients.

In the late-phase group, univariate analysis showed that none of the clinical factors (age, sex, type, and comorbidity) had significant predictive value (all *P* > 0.05; Figure S3). The RadScore demonstrated improved performance in predicting poor outcome (C-index increased from 0.743 to 0.885), and 28-day poor outcome (AUC increased from 0.793 to 0.976). This indicated that in the late-phase COVID-19, the CT image contained more prognostic information. This is consistent with the previous study that the radiological findings in the follow-up CT might be more effective in identifying the progression of COVID-19 10. The RadScore alone achieved similar performance to the CrrScore, indicating applying CT imaging alone can accurately predict poor outcome in the late-phase COVID-19 patients.

Moreover, the RadScore can successfully stratify patients with COVID-19 into low- or high-RadScore groups with significantly different survival time of poor outcome (all *P* < 0.05) in both groups. The CrrScore could also significantly stratify patients with different prognoses regarding different age, type, and comorbidities subgroups (**Figure 5, 6, and 7**). High-CrrScore patients with older age, severe type, and comorbidities had significantly higher cumulative probabilities of poor outcome. For these patients, early prevention and aggressive treatment should be administered.

Radiomics has shown potential as a non-invasive and quantitative tool in diagnosis and prognosis by extracting effective imaging features. However, some technical challenges might influence its clinical applicability in practice. One of these challenges is how to define the ROI for analysis precisely. The manual delineation is simple and effective but may result in a lack of reproducibility. Moreover, lung abnormalities in patients with COVID-19 often manifested as bilateral or multizonal lung involvement on chest CT imaging 47,48. Therefore, the 3D-ROI approach can reflect more pulmonary information. Consequently, we applied an automatic algorithm to segment the entire lung volume. This 3D-ROI can provide the entire volumetric pulmonary features and may be less influenced by manual delineation. To increase the reproducibility of the radiomics features, we conducted a low-pass Gaussian filter before voxel resampling to prevent aliasing and applied ComBat to harmonize radiomics features per scanner. Afterward, we rotated and translated both image and mask to emulate the different patient positioning, and grew and shrunk the mask volume to mimic variance in the boundaries of the 3D-ROI. The results showed that all the features in the RadScore exhibited 95% CI of ICC larger than 0.9, indicating the features in the RadScore are robust.

The five radiomics features selected in the RadScore included four texture features and one shape feature in the early-phase group, and one texture feature, two shape features, and two intensity features in the late-phase group. These may be explained by the fact that in the early-phase, most radiological findings were local pure GGO whereas in the late-phase widespread consolidation lesions were more common. In the RadScore_latephase, the 10^th^ percentile and minimum of intensity in the lung area is negatively associated with poor outcome, indicating that the more abnormal lung tissues, the worse poor outcome. This is consistent with the previous study that well-aerated lung was a negative predictor of poor outcome 27. Note that two features (shape_Sphericity and ngtdm_Complexity) overlaid in the early-phase and late-phase group, suggesting that the morphological change of lung and texture complexity were essential in predicting poor outcome no matter in early-phase or late-phase group.

To further interpret the RadScore, we did stratified analyses to explore the association between the RadScore and poor outcome regarding different symptoms and laboratory measures. Since only a part of the patients had records of symptoms and laboratory exams, we did the analyses in the combined cohort. The results demonstrated that low-RadScore and high-RadScore groups had significant differences between the patients with dyspnoea and the patients without dyspnoea on admission in the early-phase COVID-19 group (*P* = 0.004; in Table S1). The lymphocyte count demonstrated the significant difference between low- and high-RadScore subgroups in both early-phase and late-phase groups. Moreover, in the late-phase group, C-reactive protein (CRP) and creatine kinase isoenzyme MB (CK-MB) demonstrated the significant difference between low-and high-RadScore subgroups (Table S2). This indicated that RadScore could reflect the inflammatory responses, which is backed by the evidence that the majority of COVID-19 had a high-level of CRP 6. Since some research showed that SARS-COV-2 might also cause myocardial injury and chronic damage to the cardiovascular system through angiotensin-converting enzyme 2 (ACE2) receptors 49, the significant association between CK-MB and RadScore may indicate that RadScore could reveal the COVID-19-related myocardial lesion in the late-phase group. This is consistent with previous research that CK-MB had a significant difference between ICU patients and non-ICU patients 5.

Despite the favorable prognostic efficacy of the clinic-radiomics signature, our research still has some limitations. Firstly, a more extensive and prospective study cohort was needed to generalize the performance of the RadScore and CrrScore in the future. Since our models were built based on the patients who were diagnosed with COVID-19 and received chest CT, the RadScore and CrrScore are not applicable to the scenarios where the presence of the disease is unknown, such as radiological screening and population-wide (random) screening tests. In the latter case, the study cohort may include asymptomatic patients but not included in the current patient cohort. Secondly, some laboratory factors were reported associated with prognosis in COVID-19; more comprehensive clinical risk factors need to be included in the future.

In conclusion, this research proposed a non-invasive and quantitative prognostic tool for predicting poor outcome in COVID-19 based on CT imaging. Taking the insufficient medical recourse into account, our study might suggest that the chest CT radiomics signature of COVID-19 is more effective and ideal to predict poor outcome in the late-phase COVID-19 patients. For the early-phase patients, integrating radiomics signature with clinical risk factors can achieve a more accurate prediction of individual poor prognostic outcome, which enables appropriate management and surveillance of COVID-19.

## Supplementary Material

Supplementary figures and tables.

## Figures and Tables

**Figure 3:**
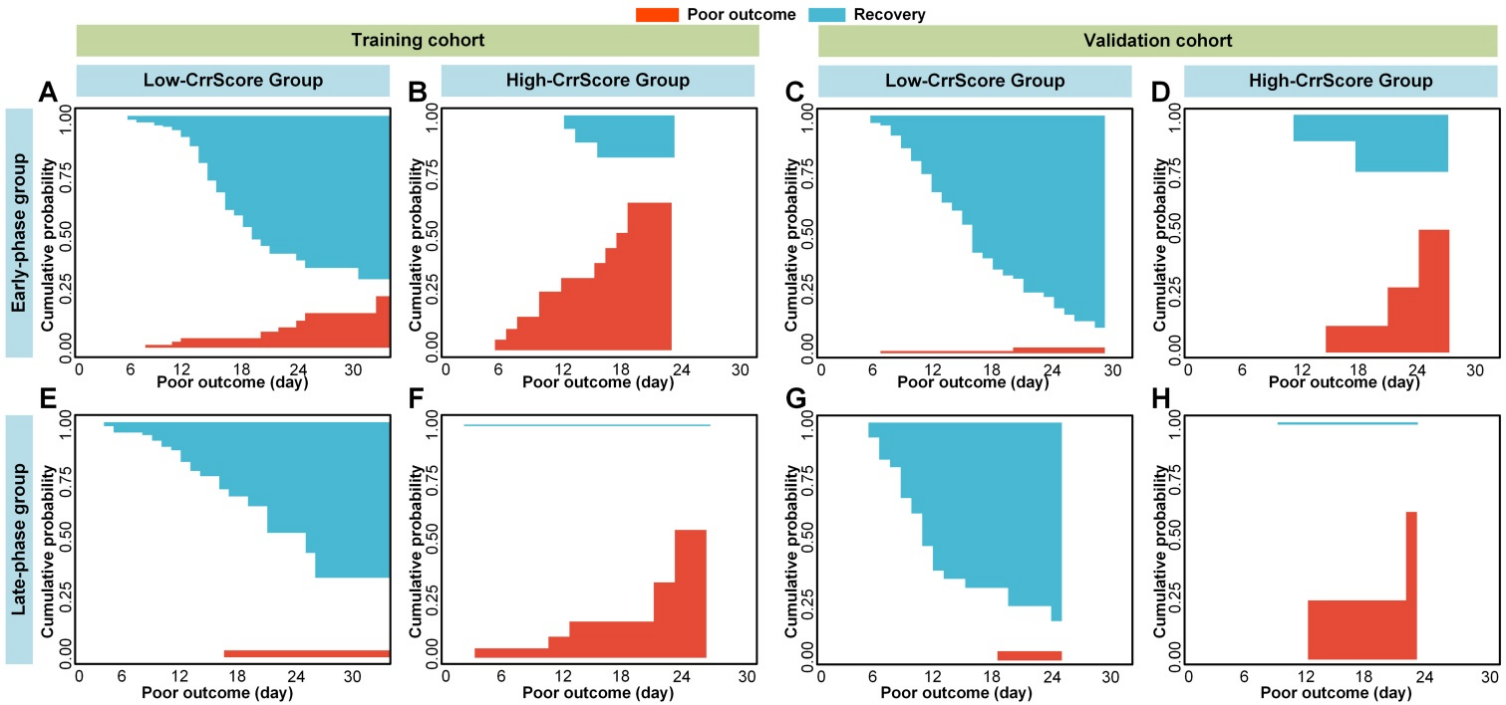
** Cumulative poor outcome and recovery probability according to the risk strata defined by CrrScore.** Red curves mean the risk of reaching to poor outcome, and the blue curves mean the risk of reaching to recovery. A), B), E), and F) in the training cohort, and C), D), G) and H) in the validation cohort. A), C), E), and G) for the low-CrrScore group, and B), D), F) and h) for the high-CrrScore group. A), B), C) and D) assess the cumulative probability in the early-phase group. E), F), G) and H) assess the cumulative probability in the late phase group.

**Table 1:** Clinical characteristics of patients with COVID-19

	Early-phase COVID-19 (n = 317)			Late-phase COVID-19 (n = 175)			Total (n=492)
Cohorts	Training (n = 212)	Validation (n = 105)	*P*	Training (n = 139)	Validation (n = 36)	*P*	
**Age, years**			0.332			0.069	
Mean (SD)	47.9(14.9)	46.1(16.3)		52.7(16.6)	46.7(17.7)		
**Sex No. (%)**			0.501			0.133	
Male	97 (45.8)	53 (50.5)		63 (45.3)	22 (61.1)		235 (47.8)
Female	115 (54.2)	52 (49.5)		76 (54.7)	14 (38.9)		257 (52.2)
**Type^a^ No. (%)**			0.829			0.161	
Mild	189 (89.2)	92 (87.6)		126 (90.6)	29 (80.6)		436 (88.6)
Severe	23 (10.8)	13 (12.4)		13 (9.4)	7 (19.4)		56 (11.4)
**Comorbidity No. (%)**							
Any	60 (28.3)	26 (24.8)	0.594	51 (36.7)	10 (27.8)	0.421	147 (29.9)
Hypertension	26 (12.3)	16 (15.2)	0.576	35 (25.2)	7 (19.4)	0.618	84 (17.1)
Cardiovascular disease	11 (5.2)	6 (5.7)	>0.99	5 (3.6)	3 (8.3)	0.444	25 (5.1)
Diabetes	22 (10.4)	3 (2.9)	0.03	7 (5.0)	3 (8.3)	0.721	35 (7.1)
Cerebrovascular disease	5 (2.4)	1 (1.0)		1 (0.7)	0 (0)		8 (1.6)
COPD	3 (1.4)	1 (1.0)		2 (1.4)	0 (0)		6 (1.2)
Pulmonary tuberculosis	4 (1.9)	0 (0)		0 (0)	0 (0)		4 (0.8)
Malignancy	6 (2.8)	2 (1.9)		4 (2.9)	1 (2.8)		13 (2.6)
Chronic kidney disease	2 (0.9)	0 (0)		2 (1.4)	0 (0)		4 (0.8)
Chronic liver disease	5 (2.4)	3 (2.9)		4 (2.9)	3 (8.3)		15 (3.0)
**Follow-up (IQR), days**							
Poor-outcome	13 (9-17)	15 (11-20)	0.017	12 (8-15)	11 (9-18)	0.235	13 (9-18)
Time interval^b^	3 (1-5)	3 (1-5)		9 (8-13)	8 (7-9)		5 (2-8)

NOTE: SD: standard deviation; IQR: interquartile range; ^a^ The type of COVID-19 is established on admission; ^b^ means the time interval between initial symptom onset and the chest CT scan on admission.

**Table 2:** Competing risk regression analysis of predictors in the training cohort

	Early-phase COVID-19			Late-phase COVID-19		
	β	HR (95% CI)	*P*	β	HR (95% CI)	*P*
Age	0.017	1.02 (0.99-1.04)	0.15	0.024	1.05 (0.99-1.11)	0.07
Comorbidity	0.231	1.26 (0.61-2.59)	0.53			
Type	2.019	7.53 (3.77-15.04)	<.001	0.888	1.38 (0.544-2.23)	0.58
RadScore	1.016	2.76 (1.76-4.32)	<.001	0.735	4.86 (1.91-12.34)	<.001
AIC	182.41			195.29		

**CrrScore_earlyphase** = 0.017 × Age + 0.231 × Comorbidity (0: Absent; 1: Present) + 2.019× Type (0: Mild Type; 1: Severe Type) + 1.016 × RadScore_earlyphase. **CrrScore_latephase** = 0.024 × Age + 0.888 × Type (0: Mild Type; 1: Severe Type) + 0.735 × RadScore_latephase.

**Table 3:** Model performance on predicting poor outcome and 28-day poor outcome probability

	Early-phase COVID-19				Late-phase COVID-19			
	C-index (95% CI)	AUC (95% CI)	SPE (%)	SEN (%)	C-index (95% CI)	AUC (95% CI)	SPE (%)	SEN (%)
**Clinical model**								
Training	0.708 (0.569-0.848)	0.748 (0.638-0.858)	63.30	79.17	0.697 (0.509-0.886)	0.648 (0.559-0.927)	89.50	50.00
Validation	0.699 (0.516-0.882)	0.685 (0.410-0.960)	59.00	80.00	0.743 (0.443-0.987)	0.793 (0.534-0.944)	96.88	75.00
**RadScore**								
Training	0.758 (0.619-0.897)	0.752 (0.631-0.873)	87.77	58.33	0.886 (0.702-0.999)	0.817 (0.693-0.999)	95.49	83.33
Validation	0.752 (0.600-0.906)	0.816 (0.614-0.999)	95.00	60.00	0.885 (0.718-0.999)	0.976 (0.906-0.999)	90.62	100.0
**CrrScore**								
Training	0.826 (0.714-0.937)	0.855 (0.740-0.920)	91.49	70.83	0.911 (0.796-0.999)	0.872 (0.687-0.999)	88.72	83.33
Validation	0.850 (0.763-0.935)	0.862 (0.680-0.999)	86.00	80.00	0.886 (0.675-0.999)	0.977 (0.932-0.999)	93.75	100.0

Note: CI: confidence interval; SPE: specificity; SEN: sensitivity; C-Index: Harrell's concordance index, and measures the performance of the poor outcome prediction. AUC: area under the receiver operating characteristic curve, and evaluates the performance of the 28-day poor outcome prediction.

## Abbreviations

COVID-19: coronavirus disease 2019
GGO: ground-glass opacity
ICU: intensive care unit
CT: computed tomography
ROI: regions of interest
C-index: concordance index
ROC: receiver-operating characteristic
AUC: area under the receiver operating characteristic curve
IQR: interquartile range
CRP: C-reactive protein
CK-MB: creatine kinase isoenzyme MB
