# Supplementary material for: Radiomics Analysis of Computed Tomography helps predict poor prognostic outcome in COVID-19
Source: Theranostics. 2020 Jun 5;10(16):7231–44. doi: 10.7150/thno.46428 (PMC7330838; doi:10.7150/thno.46428)
Supplement: Supplementary file 1 — Supplementary figures and tables. [file thnov10p7231s1.pdf]

Supplementary Material

Supplementary Methods 1. CT acquisition parameters used in the present study.

In the four involved hospitals, CT scans were performed using one of the five different scanners: Philips Brilliance Big Bore, GE Light speed VCT 4, GE Light speed Pro 16, GE Optima CT660, GE Optima CT680. All the patients underwent spiral CT scans from the lung apex to base at suspended maximum inspiration. The scans were performed at tube voltage 120 kV, tube current 200~500 mAs, rotation time 0.4~0.7 s, pixel matrix 512 × 512. Most CT scans were reconstructed with a slice thickness ≤ 2.5 mm.

Supplementary Methods 2. Details of the lung volume segmentation

To segment lung automatically from chest CT images, we built the DenseNet121-FPN model according the open-source projects in https://github.com/qubvel/segmentation_models. Here, we built an FPN model with DenseNet121, which was pre-trained in ImageNet dataset as the backbone and defined in this model as DenseNet121-FPN. Afterward, the DenseNet121-FPN model was fine-tuned using the VESSEL12 dataset [5], which includes slice-by-slice manual lung segmentation of 20 subjects. During the finetuning process, every three adjacent image slices formed a 3-channel image and were rescaled from 512×512 to 256×256 using third-order spline interpolation. We used binary cross-entropy combined with jaccard loss as loss function, and used Adam optimizer for training. The learning rate in the Adam optimizer was initialized to 0.001, and reduced by a factor of 0.5 if the loss value stop decreasing after 2 epoches. We also uploaded the fine-tuned lung segmentation model and the complete code in the github: https://github.com/wangshuocas/COVID-19

Supplementary Methods 3. The recovery and discharge criteria for patients with COVID-19

The recovery and discharge criteria were: normal body temperature for greater than 3 days, and significantly improved respiratory symptoms, and significantly improved exudative lesions through radiological evaluation, and two consecutive negative nucleic acid detection with at least 24 hours apart. (National Health Commission of the People's Republic of China. Diagnosis and treatment protocols of pneumonia caused by a novel coronavirus (trial version 6))

Supplementary Methods 4. Definition of the features in the signature

1. **shape_Sphericity:**

   Sphericity is a measure to describe how sphere-like the volume is:

$$sphericity = \frac{\sqrt[3]{36\pi V^2}}{A}$$

   Here, V is the volume; A is the surface area.

2. **shape_Flatness:**

   The ratio of the major and least axis lengths could be viewed as the extent to which a volume is flat relative to its length. For computational reasons, *flatness* is defined as an inverse ratio. 1 is thus completely non-flat, e.g. a sphere, and smaller values express objects which are increasingly flatter.

$$Flatness = \sqrt{\frac{\lambda_{least}}{\lambda_{major}}}$$

   Here, λ_{least} and λ_{major} are the lengths of the largest and smallest principal component axes. The values range between 1 (non-flat, sphere-like) and 0 (a flat object, or single-slice segmentation).

3. **firstorder_Minimum**:

   The minimum intensity value in $\boldsymbol{X}$, i.e. $\min \boldsymbol{X}$. Here, $\boldsymbol{X}$ is is a set of N_p voxels included in the ROI.

4. **firstorder_10Percentile:**

   10Percentile is the 10th percentile of $\boldsymbol{X}$**.** Here, $\boldsymbol{X}$ is a set of N_p voxels included in the ROI.

5. **glcm_ClusterShade:**

   A Gray Level Co-occurrence Matrix (GLCM) of size $N_g \times N_g$ describes the second-order joint probability function of an image region constrained by the mask and is defined as $\boldsymbol{P}_{\boldsymbol{\alpha},\boldsymbol{\delta}}(i,j)$. The $(i,j)^{th}$ element of this matrix represents the number of times the combination of levels i and j occur in two pixels in the image, that are separated by a distance of *δ* pixels along angle *θ*. The distance *δ* from the center voxel is defined as the distance according to the infinity norm.

$$cluster\ shade = \sum_{i=1}^{N_g}\sum_{j=1}^{N_g}\left(i + j - \mu_x - \mu_y\right)^3 p(i,j)$$

Here, N_g is the number of discrete intensity levels in the image; $\boldsymbol{P}(i,j)$ is the co-occurrence matrix for an arbitrary δ and θ; $p(i,j)$ is the normalized co-occurrence matrix and equal to $\frac{\boldsymbol{P}(i,j)}{\sum \boldsymbol{P}(i,j)}$; $p_x(i) = \sum_{i=1}^{N_g} P(i,j)$ is the marginal row probabilities; $p_y(i) = \sum_{j=1}^{N_g} P(i,j)$ is the marginal column probabilities; μ_x is the mean gray level intensity of p_x and defined as $\mu_x = \sum_{i=1}^{N_g} p_x(i)i$; μ_y is the mean gray level intensity of p_y and defined as $\mu_y = \sum_{j=1}^{N_g} p_y(j)j$.

6. **glcm_Correlation:**

$$correlation = \frac{\sum_{i=1}^{N_g} \sum_{j=1}^{N_g} p(i,j)ij - \mu_x\mu_y}{\sigma_x(i)\sigma_y(j)}$$

Here, N_g is the number of discrete intensity levels in the image; $\boldsymbol{P}(i,j)$ is the co-occurrence matrix for an arbitrary δ and θ; $p(i,j)$ is the normalized co-occurrence matrix and equal to $\frac{\boldsymbol{P}(i,j)}{\sum \boldsymbol{P}(i,j)}$; $p_x(i) = \sum_{i=1}^{N_g} P(i,j)$ is the marginal row probabilities; $p_y(i) = \sum_{j=1}^{N_g} P(i,j)$ is the marginal column probabilities; μ_x is the mean gray level intensity of p_x and defined as $\mu_x = \sum_{i=1}^{N_g} p_x(i)i$; μ_y is the mean gray level intensity of p_y and defined as $\mu_y = \sum_{j=1}^{N_g} p_y(j)j$; σ_x is the standard deviation of p_x; σ_y is the standard deviation of p_y.

7. **glrlm_LongRunHighGrayLevelEmphasis (glrlm_LRHGLE):**

A Gray Level Run Length Matrix (GLRLM) quantifies gray level runs, which are defined as the length in number of pixels, of consecutive pixels that have the same gray level value. In a gray level run length matrix $\boldsymbol{P}(i,j|\theta)$, the $(i,j)^{th}$ element describes the number of runs with gray level i and length j occur in the image (ROI) along angle θ.

Here, N_g is the number of discreet intensity values in the image; N_r is the number of discrete run lengths in the image; N_p is the number of voxels in the image; N_r is the number of discreet run lengths in the image; $N_r(\theta)$ is the number of runs in the image along angle θ, which is equal to $\sum_{i=1}^{N_g} \sum_{j=1}^{N_g} \boldsymbol{P}(i,j|\theta)$; $\boldsymbol{P}(i,j|\theta)$ is the run length matrix for an arbitrary direction θ; $p(i,j|\theta)$ is the normalized run length matrix, defined as $p(i,j|\theta) = \frac{\boldsymbol{P}(i,j|\theta)}{N_r(\theta)}$.

$$LRHGLE = \frac{1}{N_r(\theta)} \sum_{i=1}^{N_g} \sum_{j=1}^{N_r} \boldsymbol{P}(i,j|\theta) i^2 j^2$$

8. ngtdm_Complexity:

A Neighboring Gray Tone Difference Matrix (NGTDM) quantifies the difference between a gray value and the average gray value of its neighbors within distance δ. The sum of absolute differences for gray level i is stored in the matrix. Let $\boldsymbol{X}_{gl}$ be a set of segmented voxels and $x_{gl}(j_x, j_y, j_z) \in \boldsymbol{X}_{gl}$ be the gray level of a voxel at position (j_x, j_y, j_z), then the average gray level of the neigbourhood is:

$$\bar{A}_i = \bar{A}(j_x, j_y, j_z) = \frac{1}{W} \sum_{k_x=-\delta}^{\delta} \sum_{k_y=-\delta}^{\delta} \sum_{k_z=-\delta}^{\delta} x_{gl}(j_x + k_x, j_y + k_y, j_z + k_z)$$

where $(k_x, k_y, k_z) \neq (0,0,0)$ and $x_{gl}(j_x + k_x, j_y + k_y, j_z + k_z) \in \boldsymbol{X}_{gl}$

Here, W is the number of voxels in the neighbourhood that are also in $\boldsymbol{X}_{gl}$.

$$Complexity = \frac{1}{N_{v,p}} \sum_{i=1}^{N_g} \sum_{j=1}^{N_g} \frac{|\, i - j \,|\, (p_i s_i + p_j s_j)}{p_i + p_j}, \text{where } p_i \neq 0, p_j \neq 0$$

Table S1. The symptom and sign of patients according to the RadScore in the combined cohorts

	Early-phase COVID-19			Late-phase COVID-19		
	Low-RadScore	High-RadScore	*P*	Low-RadScore	High-RadScore	*P*
Fever on admission			0.96			>0.99
Absent	38/192 (16.8%)	4/17 (23.5%)		4/45 (8.9%)	1/5 (20.0%)	
Present	154/192 (80.2%)	13/17 (76.5%)		41/45 (91.1%)	4/5 (80.0%)	
Cough on admission			0.68			0.22
Absent	72/191 (37.7%)	5/17 (29.4%)		18/45 (40.0%)	4/5 (80.0%)	
Present	119/191 (62.3%)	12/17 (70.6%)		27/45 (60.0%)	1/5 (20.0%)	
Myalgia or fatigue on admission			0.71			0.34
Absent	120/191 (62.8%)	12/17(70.6%)		31/45 (68.9%)	5/5 (100.0%)	
Present	71/191 (37.2%)	5/17 (29.4%)		14/45 (31.1%)	0/5 (0.0%)	
Dyspnea on admission			0.004			0.19
Absent	179/191 (93.7%)	12/17 (70.6%)		41/45 (91.1%)	3/5 (60.0%)	
Present	12/191 (6.3%)	5/17 (29.4%)		4/45 (8.9%)	2/5 (40.0%)	
Highest temperature during hospitalization			0.79			0.80
	37.90 (0.81)	37.97 (0.95)		37.04 (0.81)	37.85 (0.79)	

Table S2. The laboratory exams of patients according to the RadScore in the combined cohorts

	Early-phase COVID-19			Late-phase COVID-19		
	Low-RadScore	High-RadScore	*P*	Low-RadScore	High-RadScore	*P*
White-cell count, × 10^9 per L	5.19(2.71)	6.20(4.47)	0.57	5.19(1.98)	8.75(6.19)	0.28
Neutrophil count, × 10^9 per L	4.52(8.94)	3.86(2.09)	0.30	3.03(1.02)	6.32(6.76)	0.87
Lymphocyte count, × 10^9 per L	1.45(3.23)	0.89(0.51)	0.003	1.22(0.57)	0.67(0.30)	0.02
Hemoglobin, g/L	163(73)	164(82)	0.53	168(68)	128 (16)	0.19
Platelet count, × 10^9 per L	203(85)	178(71)	0.53	184(44)	192 (75)	0.91
Albumin, g/L	42.7(37.7)	32.4(5.9)	0.002	38.3(3.6)	34.8(6.1)	0.33
ALT, U/L	32(27)	34(17)	0.22	29(17)	42 (35)	0.74
AST, U/L	33(25)	41(21)	0.09	34(18)	32 (6)	0.66
Total bilirubin, mmol/L	10(6)	27(26)	0.08	10.1(3.8)	20.1(19.0)	0.74
Creatinine, µmol/L	71(31)	105(105)	0.40	71(25)	80 (34)	0.63
Creatine kinase, U/L	113(116)	294(557)	0.43	138(166)	232 (175)	0.39
CK-MB, U/L	160(143)	251(246)	0.17	127(165)	73(137)	0.02
Troponin I, ng/mL	0.22(0.76)	0.07(0.08)	0.27	0.06(0.05)	0.21(0.97)	0.07
C-reactive protein, mg/L	27(35)	39(47)	0.43	22 (26)	57 (65)	0.03
Procalcitonin, ng/mL	0.18(0.35)	0.16(0.10)	0.12	0.15(0.27)	0.25(0.43)	0.43

NOTE: ALT: Alanine aminotransferase; AST: Aspartate aminotransferase; CK-MB: Creatine kinase isoenzyme MB

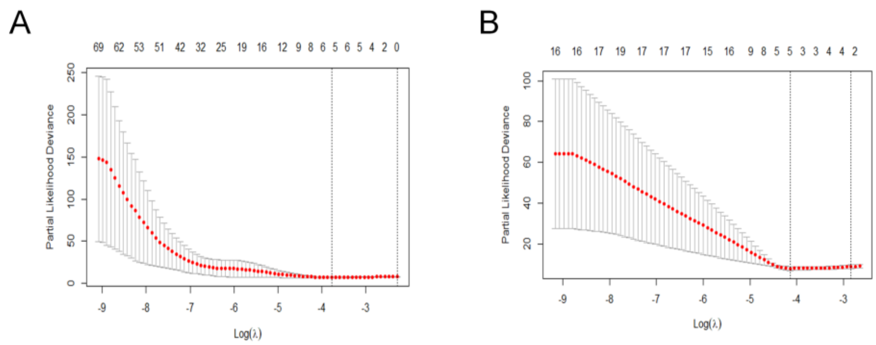

Figure S1. Radiomics feature selection using the least absolute shrinkage and selection operator (LASSO) method. Tuning parameter (λ) selection in the LASSO model used 10-fold cross-validation via minimum criteria. The partial likelihood deviance (PLD) curve was plotted versus log (λ). Dotted vertical lines were drawn at the optimal values by using the minimum criteria and 1 standard error of the minimum criteria (the 1-SE criteria). A) LASSO in the early-phase COVID-19 patients; B) LASSO in the late-phase COVID-19 patients.

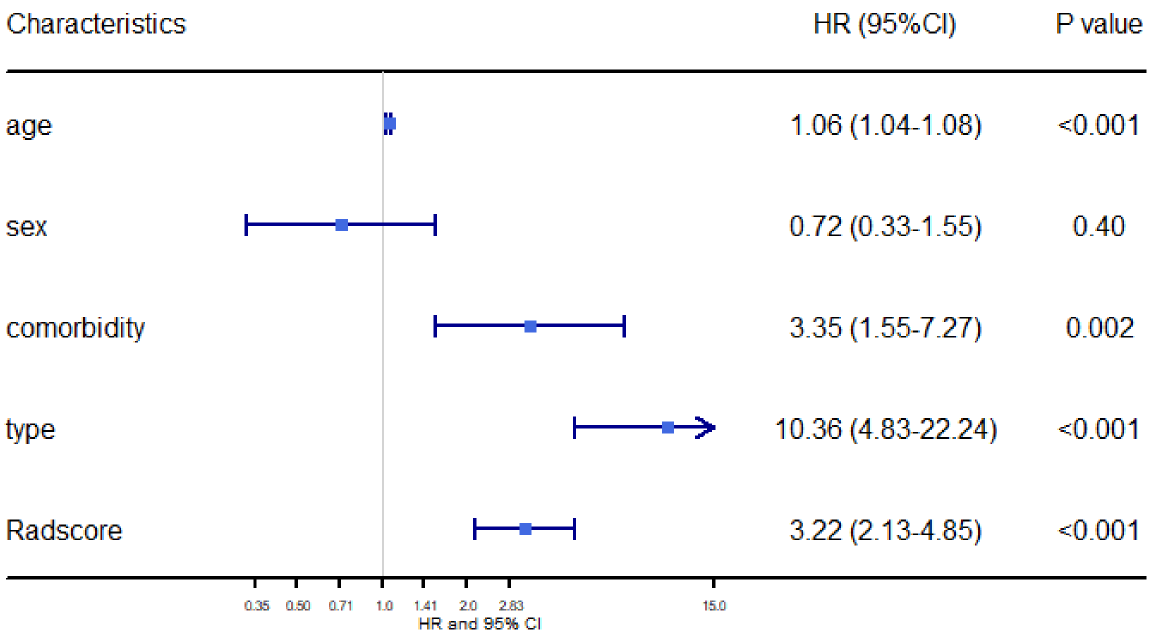

Figure S2. Forest plot of potential predictors for poor outcome in the early-phase COVID-19 patients by univariate analysis in the training cohort.

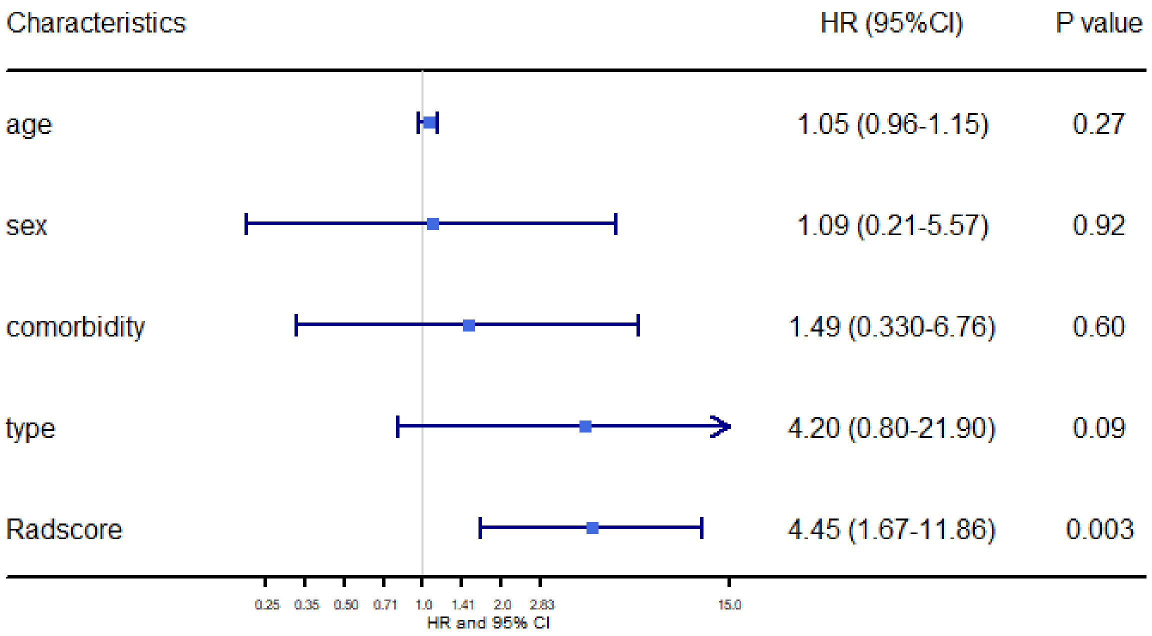

Figure S3. Forest plot of potential predictors for poor outcome in the late-phase COVID-19 patients by univariate analysis in the training cohort.

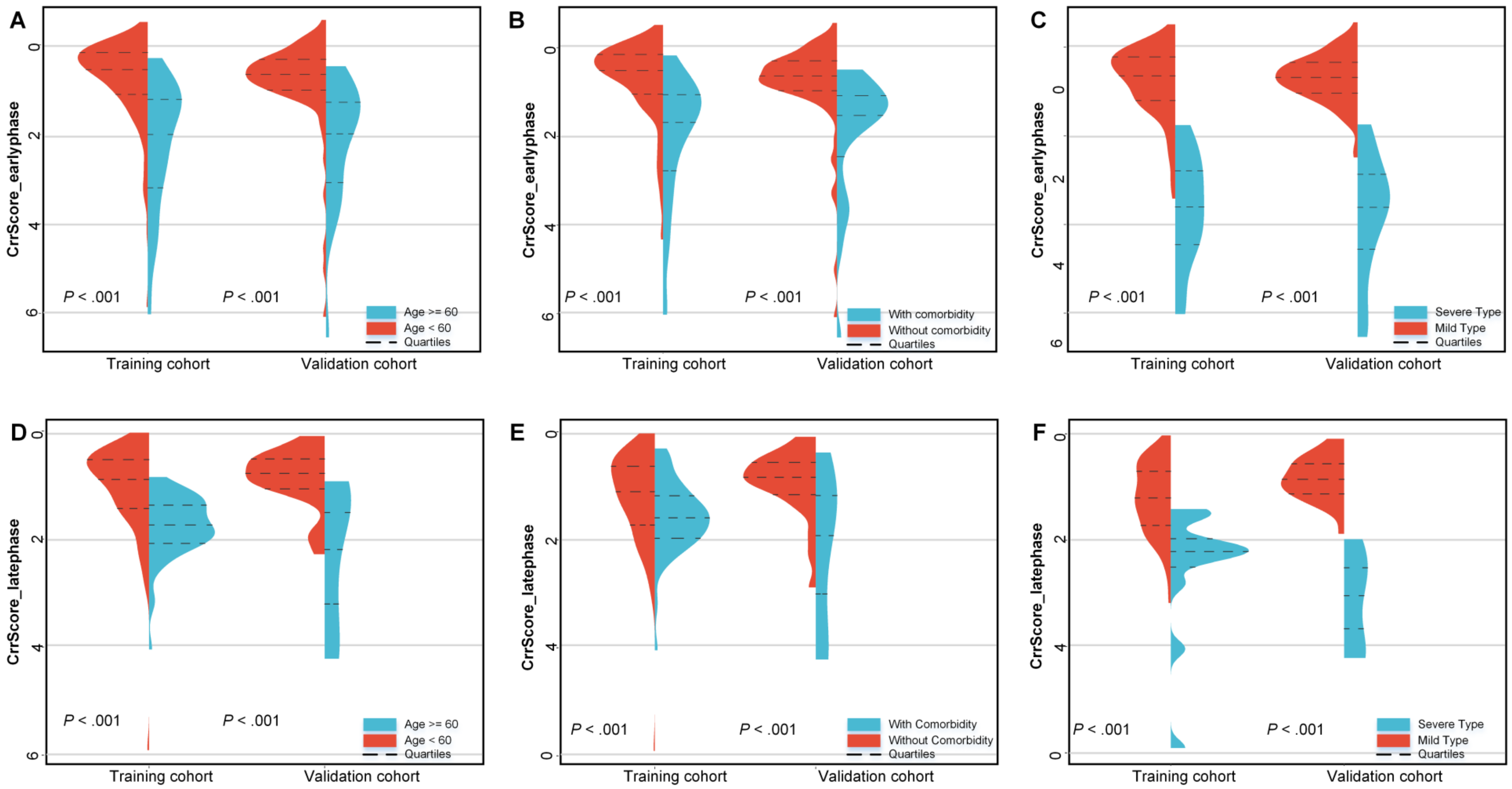

Figure S4. The distribution of Crrscore_earlyphase and CrrScore_latephase regarding different the age, type, and comorbidity subgroups. A) and D) CrrScore between age<60 and age>=60 subgroups; B) and E) CrrScore between patients with and without comorbidity; C) and F) CrrScore between mild and severe type COVID-19 patients. A), B), and C) assess the CrrScore in the early-phase COVID-19 patients. D), E), and F) assess the CrrScore in the late-phase COVID-19 patients.

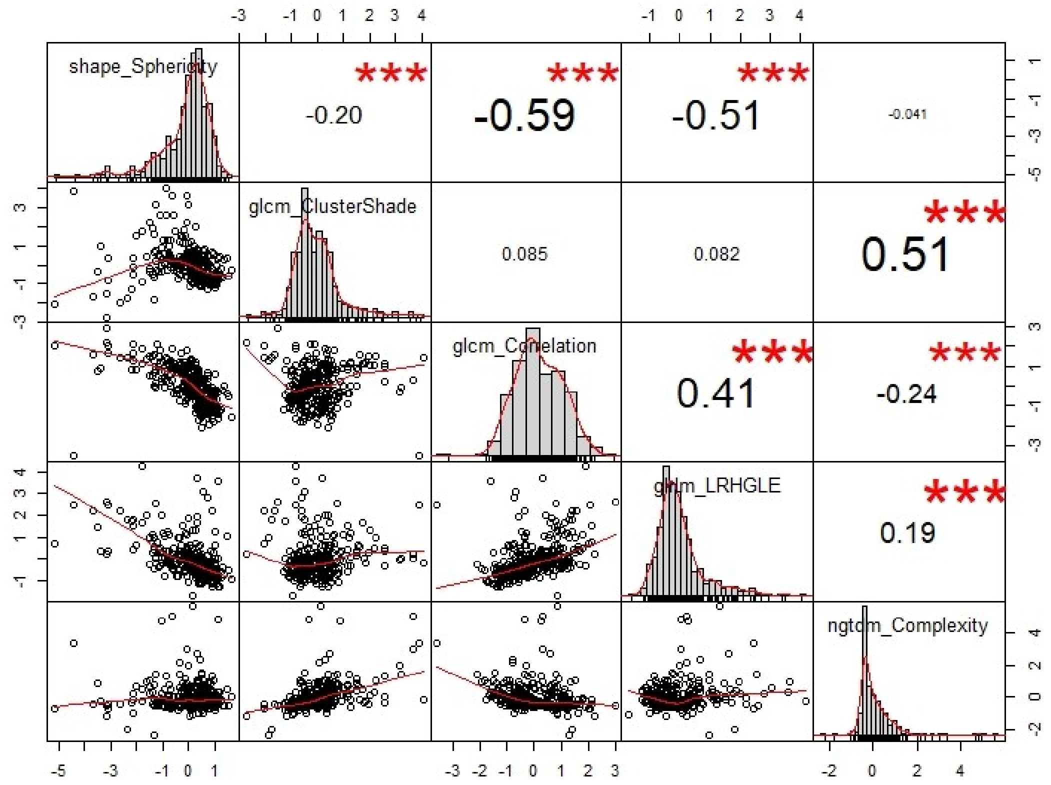

Figure S5. Correlation plot of the five features in the signature Radscore_earlyphase. The histograms of the five features (shape_Sphericity, glcm_ClusterShade, glcm_Correlation, glrlm_LRHGLE, and ngtdm_Complexity) are shown on the diagonal. The bivariate scatter plots with a fitted line are displayed on the bottom of the diagonal. The value of the correlation plus the significance level as stars on the top of the diagonal. The asterisks indicate the significance levels of the correlations, * $p < 0.05$, ** $p < 0.01$, *** $p < 0.001$.

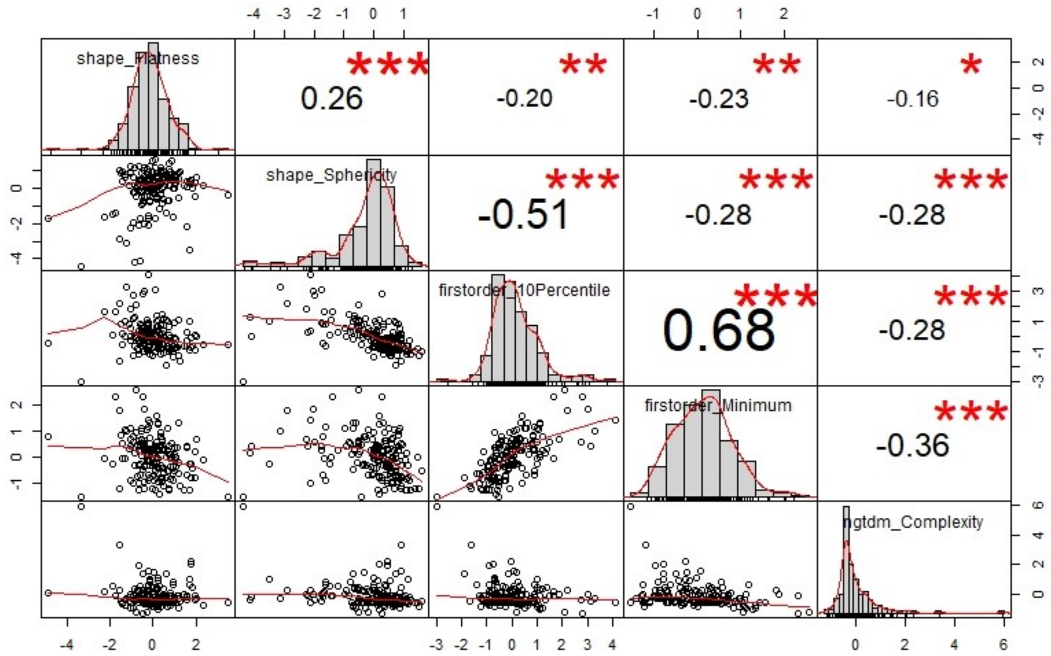

Figure S6. Correlation plot of the five features in the signature Radscore_latephase. The histograms of the five features (shape_Flatness, shape_Sphericity, firstorder_10Percentile, firstorder_Minimum, and ngtdm_Complexity) are shown on the diagonal. The bivariate scatter plots with a fitted line are displayed on the bottom of the diagonal. The value of the correlation plus the significance level as stars on the top of the diagonal. The asterisks indicate the significance levels of the correlations, * $p < 0.05$, ** $p < 0.01$, *** $p < 0.001$.

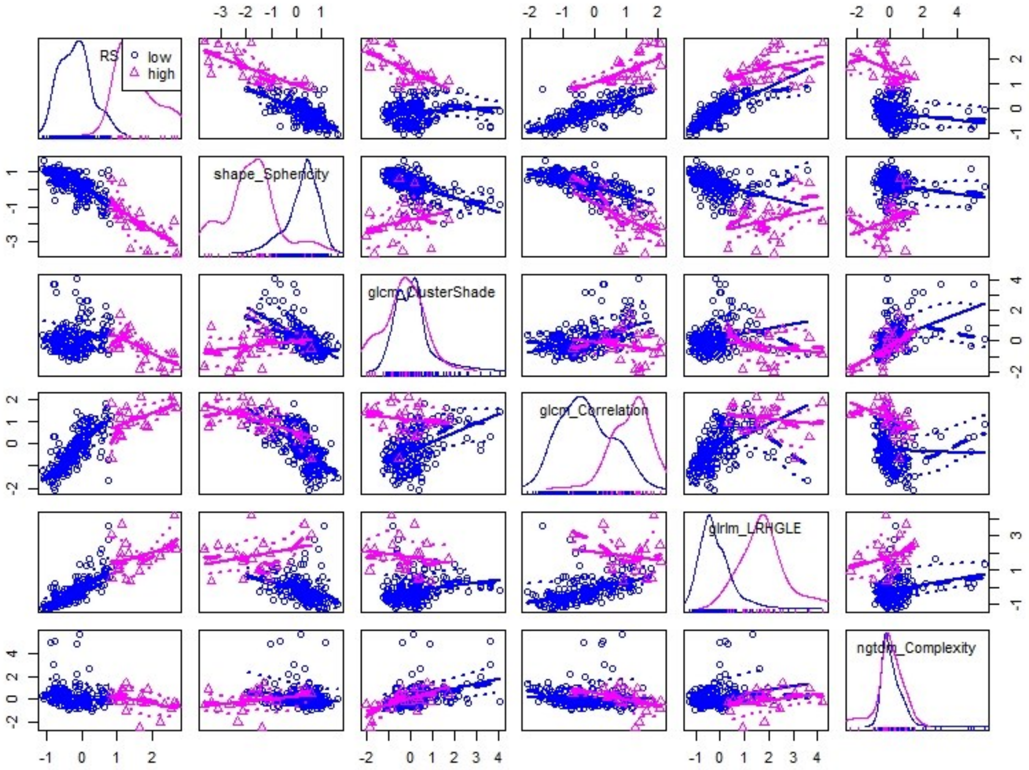

Figure S7. Scatterplot matrix of the interrelationship between RadScore_earlyphase and the five features in the signature RadScore_earlyphase in the training cohort. RS: RadScore_earlyphase.

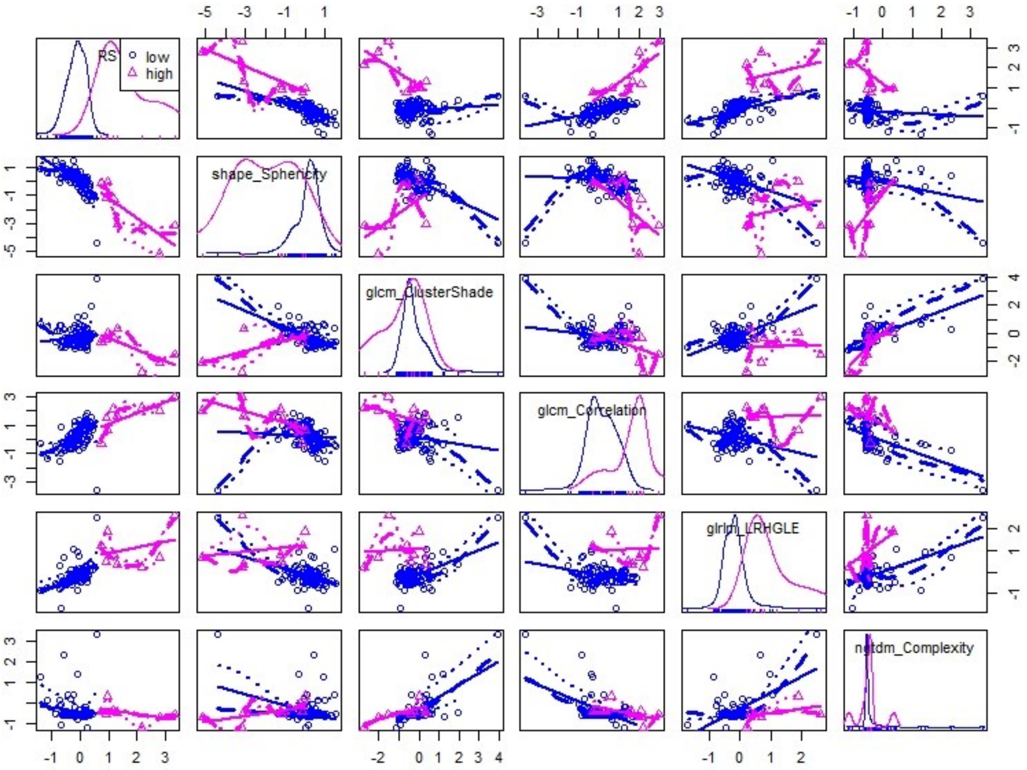

Figure S8. Scatterplot matrix of the interrelationship between RadScore_earlyphase and the five features in the signature RadScore_earlyphase in the validation cohort. RS: RadScore_earlyphase.

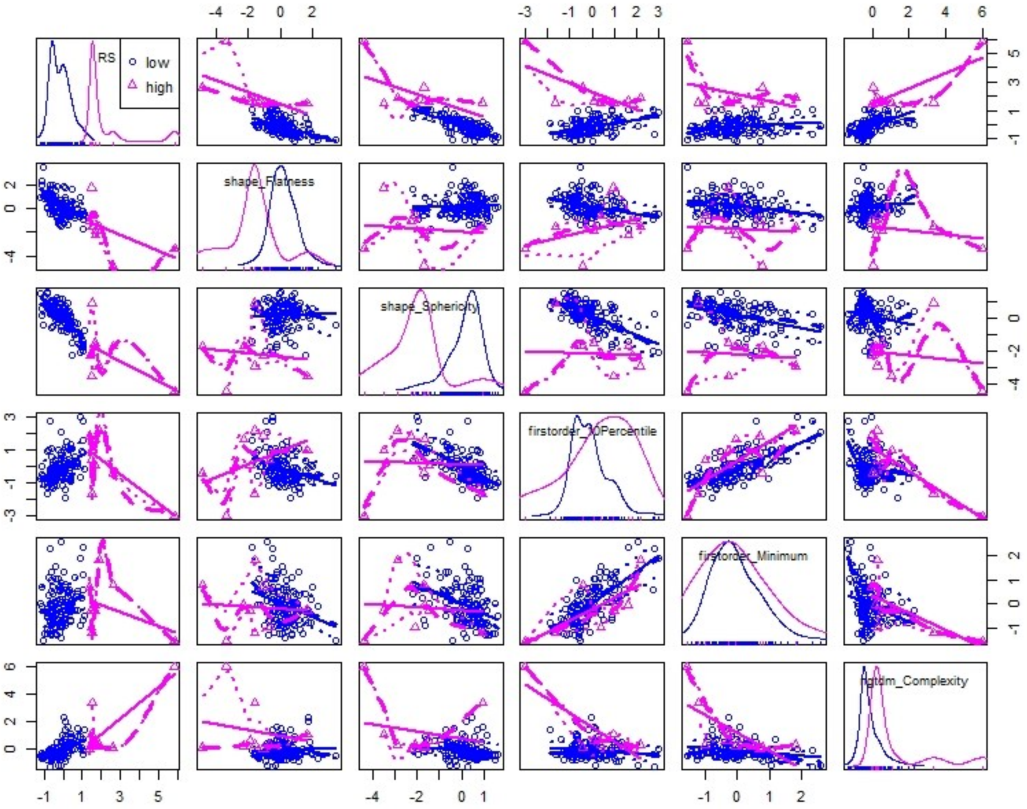

Figure S9. Scatterplot matrix of the interrelationship between RadScore_latephase and the five features in the signature RadScore_latephase in the training cohort. RS: RadScore_latephase.

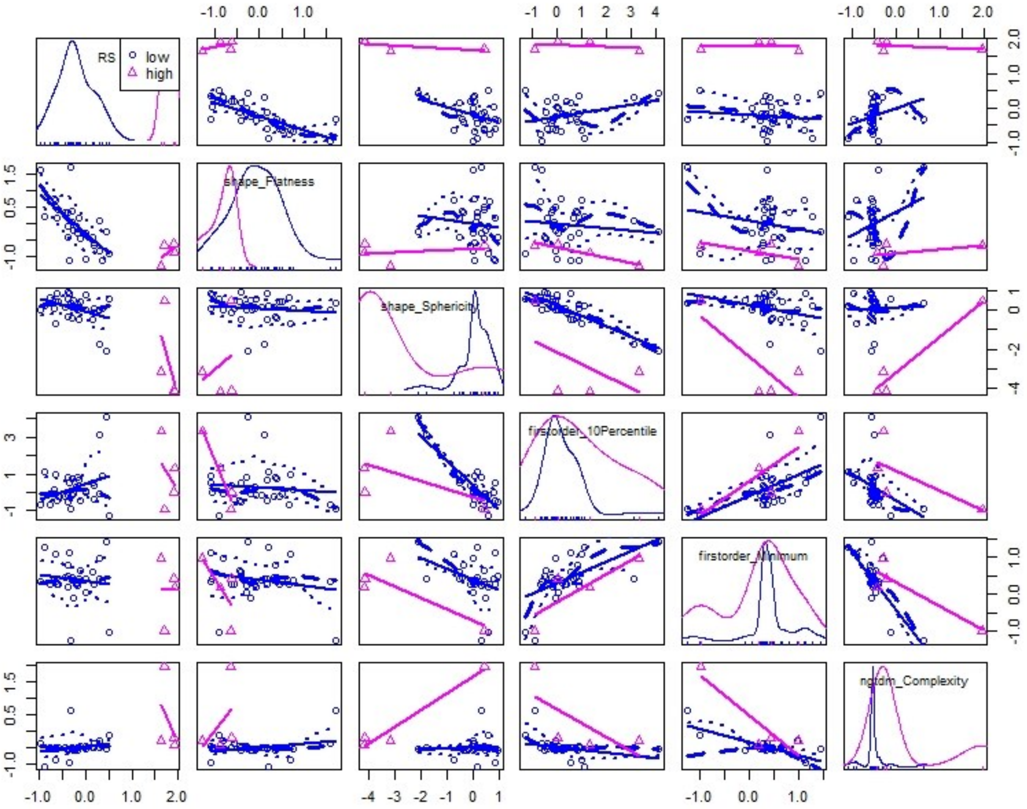

Figure S10. Scatterplot matrix of the interrelationship between RadScore_latephase and the five features in the signature RadScore_latephase in the validation cohort. RS: RadScore_latephase.

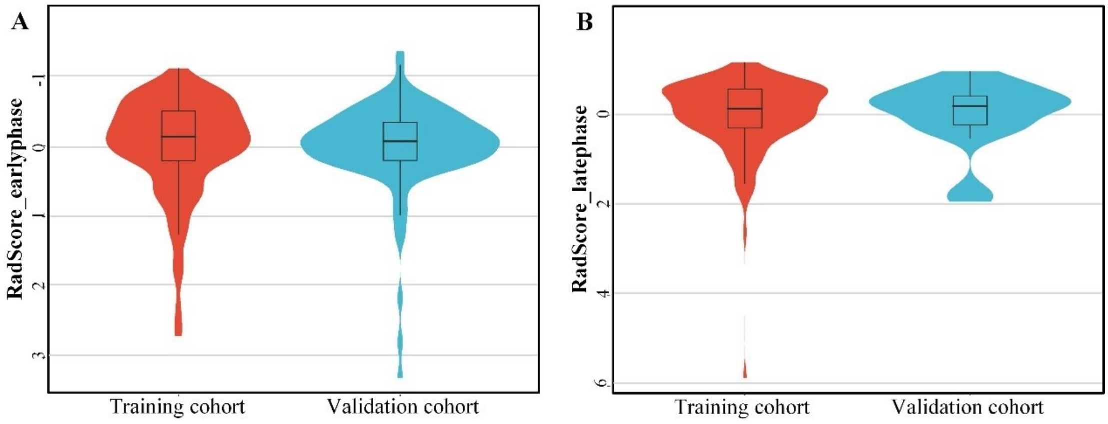

Figure S11. The distribution of RadScore_earlyphase and RadScore_latephase.
